# Appendiceal intussusception requiring an ileocecectomy: a case report and comment on the optimal surgery

**DOI:** 10.1186/s12893-018-0380-9

**Published:** 2018-08-01

**Authors:** Byung-Soo Park, Dong Hoon Shin, Dong-il Kim, Gyung Mo Son, Hyun Sung Kim

**Affiliations:** 1 Department of Surgery, Pusan National University Yangsan Hospital, 20 Geumo-ro, Mulgeum-eup, Yangsan, Gyungsangnam-do 50612 Republic of Korea; 2 Department of Pathology, Pusan National University Yangsan Hospital, Yangsan, Republic of Korea

**Keywords:** Intussusception, Appendix, Appendiceal neoplasm, Ileocecectomy, Laparoscopic surgery

## Abstract

**Background:**

Appendiceal intussusception is very rare condition with an estimated incidence of 0.01%. Therefore, it is likely to be overlooked. In addition, making the diagnosis before or during surgery is very difficult.

**Case presentation:**

A 60-year-old male who was referred to our gastroenterology center with cecal inflammation found during a colonoscopy. An abdominal computed tomography (CT) following endoscopy revealed a 5 × 2.5 × 4 cm mass-like lesion in the cecum around the ileocolic (IC) valve and appendiceal orifice. The main lesion seemed to be an inflammatory mass rather than a malignancy because it appeared to be an extraluminal or extramucosal lesion. Ultrasonography revealed diffuse wall thickening of the cecum around the appendiceal orifice that was suspicious for an inflammatory mass or a benign mass. A diagnosis was uncertain. The differential diagnosis included chronic appendicitis, appendiceal neoplasm such as appendiceal mucocele, low grade appendiceal mucinous neoplasm. The patient underwent a laparoscopic partial cecectomy. In the surgical field, there was a large mass in the appendiceal orifice. The cecum was partially resected, with care taken to preserve the IC valve. Final histopathological analysis of the surgical specimen revealed an appendiceal intussusception without any mucosal lesion of the appendix. Narrowing of the terminal ileum with a small bowel obstruction and stenosis of the IC valve occurred postoperatively. Therefore, ileocecectomy was performed via a laparoscopic approach. The patient was discharged 11 days after the second surgery without another significant postoperative complication.

**Conclusions:**

We report a rare case of appendiceal intussusception that required reoperation due to ileocolic valve stenosis. If the correct diagnosis of appendiceal intussusception is made, we can select an appropriate surgical treatment based on the classification of appendiceal intussusceptions.

## Background

The appendiceal diseases that require surgery are mostly appendicitis or appendiceal neoplasms, such as appendiceal mucoceles. Appendiceal intussusception is quite rare and is likely to be overlooked [1]. The incidence is approximately 0.01% [2]. Preoperative diagnosis is accomplished with computed tomography (CT) or ultrasonography, but the diagnosis is very difficult, and many cases are diagnosed during or after surgery [1, 3]. Due to the difficulty of diagnosis and the rarity of this disease, the optimal treatment is still unclear [4]. We report a case of appendiceal intussusception who required laparoscopic ileocecectomy and discuss the optimal surgery for it.

## Case presentation

A 60-year-old male was referred to our center with cecal inflammation found during a screening colonoscopy. He did not complain of any abdominal discomfort, such as pain, nausea, vomiting, and diarrhea. He had no past medical history except surgery for an inguinal hernia. He was afebrile with stable vital signs. On a physical examination, there was no tenderness in the abdomen.

**Fig. 1:**
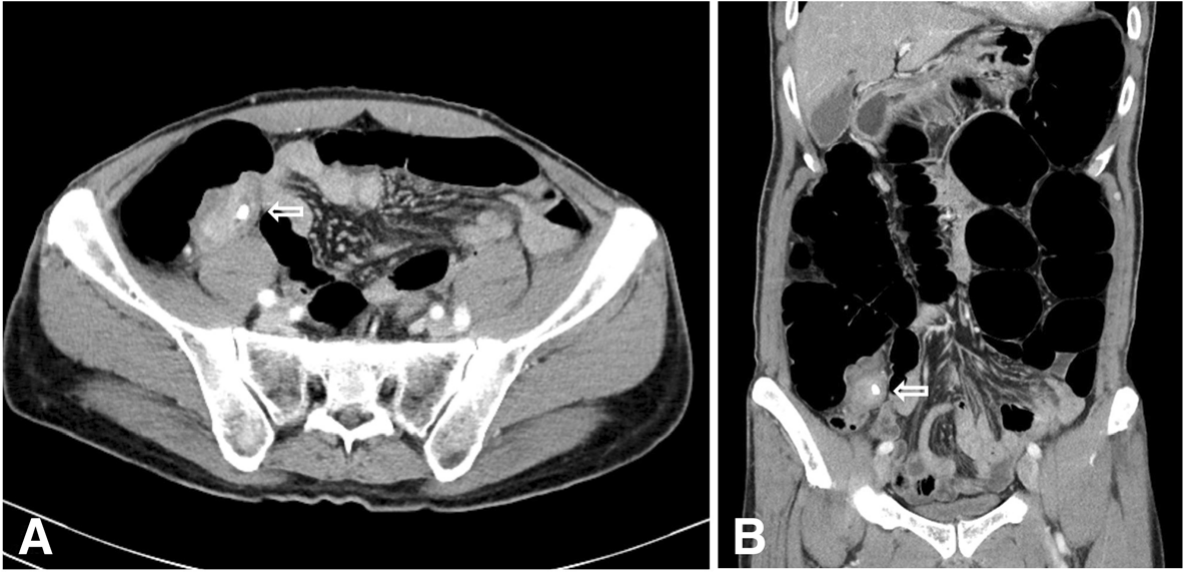
CT abdomen & pelvis images. The images show a 5 × 2.5 × 4 cm mass-like lesion in the cecum. Transverse view (**a**). Coronal view (**b**)

Colonoscopy performed at the local clinic revealed a hyperemic inflammatory lesion in the cecum around the appendiceal orifice. Because the lesion felt very hard during the colonoscopic biopsy, it was likely associated with a long period of inflammation. An abdominal CT performed after the colonoscopy in the clinic revealed a 5 × 2.5 × 4 cm mass-like lesion in the cecum around the ileocolic (IC) valve and appendiceal orifice; it had heterogenous enhancement and an ovoid calcification (8 mm) at its center, which was suspected to be an appendicolith (Fig. 1). The lesion was accompanied by appendicitis, which was identified based on appendiceal dilation (9 mm) and haziness of the periappendiceal fat. The main lesion seemed to be an inflammatory mass rather than a malignancy because it appeared to be an extraluminal or extramucosal lesion. Ultrasonography revealed a diffuse area of wall thickening (4.9 × 2 × 2.5 cm) in the cecum around the appendiceal orifice that was suspicious for an inflammatory mass or benign mass. The 8-mm calcification identified on the previous CT was probably in the mass-like wall thickening rather than in the appendix. The appendiceal lesion seemed to be a mucocele rather than acute appendicitis because there was no periappendiceal inflammation, a thin wall, and no direct tenderness. Colonoscopic biopsy of the cecum showed mild chronic nonspecific colitis with mucosal lymphoid follicles.

**Fig. 2:**
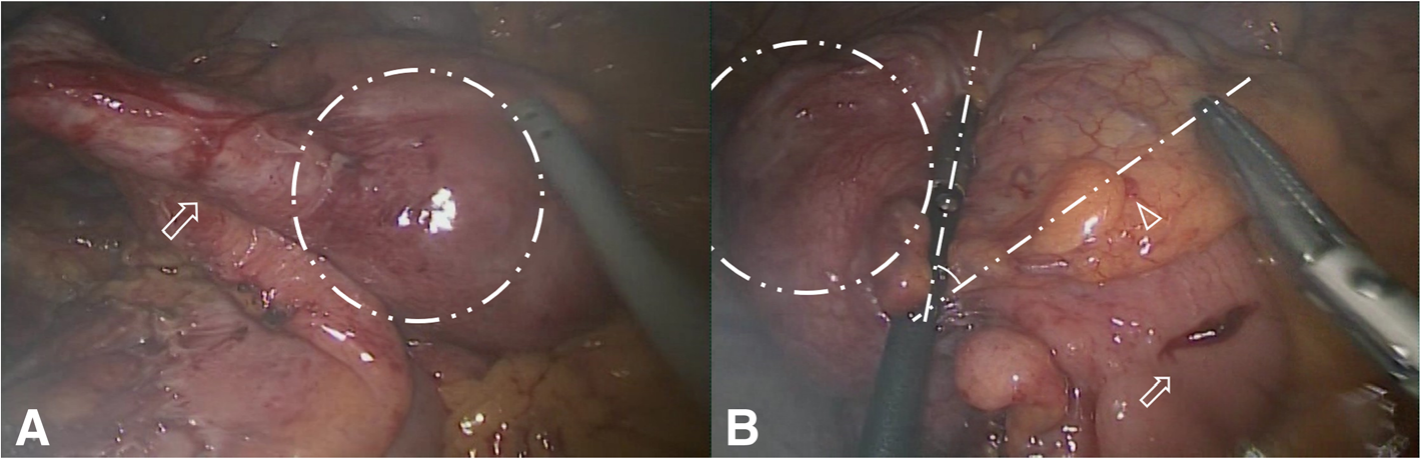
Surgical findings. A large mass is present in the appendiceal orifice. There does not appear to be an intussusception in the surgical field. First image shows the appendix and the cecum. Appendix (arrow), mass formed by the intussusception (dotted circle) (**a**). Second image shows the ileum and the cecum. Ileum (arrow), IC valve (arrowhead), mass formed by the intussusception (dotted circle), lines with IC valve and edge of the mass (dotted line) (**b**)

**Fig. 3:**
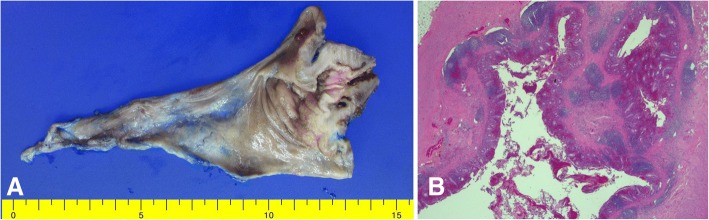
Histopathological findings. Gross findings show an appendiceal intussusception (**a**). Pathologic findings demonstrate no mucosal lesions of the appendix (H&E, 20×) (**b**)

The patient underwent laparoscopic partial cecectomy. In the surgical field, there was a large mass in the appendiceal orifice (Fig. 2). There did not appear to be an intussusception involving the appendix or other intestine including the ileum. The cecum was partially resected, with care taken to preserve the IC valve. After the resection, it was thought that the ileocolic valve had enough remaining lumen to allow the passage of bowel contents. Final histopathological analysis of the surgical specimen revealed an appendiceal intussusception without any mucosal lesion of the appendix (Fig. 3). Three days postoperatively, the patient had abdominal distension without gas passage. An X-ray revealed a small bowel obstruction pattern. An L-tube was inserted, and the patient remained NPO. Despite conservative management, the symptoms were ongoing, and an abdominal CT was performed 6 days after surgery. This CT showed abrupt narrowing of the terminal ileum with a small bowel obstruction and stenosis of the IC valve. Emergency laparoscopic exploration was performed. In the surgical field, small bowel dilatation was present just proximal to the IC valve and raised concerns for IC valve stenosis. Therefore, a laparoscopic ileocecectomy was performed. The patient was discharged 11 days after the second surgery without a significant postoperative complication. We carried out the follow-ups regularly with CT, and he remained asymptomatic for 2 years postoperatively.

## Discussion and conclusions

Appendiceal intussusception is very rare. Since the first report on the intussusception by McKidd in 1858, the literature on it has been confined to a few case reports and small cases series [1, 5]. Thus, there are no clear guidelines for the management of this disease [4]. In particular, since this disease is very difficult to diagnose preoperatively, many cases are identified during or after surgery [1, 6]. The inacuracies of a preoperative diagnosis can put surgeons through unsuspected difficulties [1].

Ultrasonography plays an important role as a diagnostic method for appendiceal intussusceptions in children [1, 7]. Abdominal CT is the most commonly used testing method in adults. The presence of a concentric central mass (target-like sign) can be helpful for diagnosis [7]. However, even though we reviewed it again, there was no doubt about an appendiceal intussusception based on CT and ultrasonography. In this case, the bowel wall continued to undergo inflammation and fibrosis due to repeated intussusception, resulting in the formation of a mass-like appearance.

If the intussusception is misdiagnosed as an appendiceal neoplasm before or during surgery, such as this case, treatment is performed as if the lesion is an appendiceal neoplasm. This is because we need to check first if it is malignant. Therefore, it is important to remove the suspicious part of the tumor and perform an accurate pathologic examination. An appendectomy can be performed if the lesion can be resected completely. A partial cecectomy should be performed if clear resection margins cannot be achieved because the mass lesion involves the cecum [8]. If the possibility of ileocecal stenosis after resection is suspected owing to the proximity of the mass to the ileocecal valve, performing ileocecectomy during the initial surgery is advisable.

However, the possibility of stenosis is sometimes ambiguous as in the present case. If open surgery is performed, the diameter and preservation of the IC valve can be confirmed with fingers. However, this is technically limited in laparoscopic surgery. Here, we can consider two methods. First, the angle between the resection line and the IC valve can be checked, regardless of whether the distance between them is sufficient. On the contrary, if the cecum should be resected close to the ileocecal valve because of close proximity of the mass, the angle between the resection line and the ileocecal valve could be meaningful. This does not pose any problems if the predicted angle is > 90°. However, partial cecectomy at an angle of < 45° might confer a risk of stenosis or congestion. The angle was approximately 40° in this case (Fig. 2). Further research is needed for clearer conclusions. Second, valve preservation and the internal diameter can be examined with intraoperative colonoscopy just after the cecectomy. It can be strongly recommended as the most definite method for cases with a risk of stenosis.

Ileocecectomy should be considered if stenosis is suspected. However, if the neoplasm is revealed to have malignant potential in the pathological report, a right hemicolectomy may be considered in the future [8, 9]. Therefore, we should be more prudent in this area. If malignancy is strongly suspected before surgery, a right hemicolectomy may reduce the risk of reoperation or tumor seeding compared with an ileocecectomy. As such, even if the lesion is ultimately determined to be appendiceal intussusception, surgeries that are performed as if the lesion is an appendiceal or cecal tumor are inevitable in cases of appendiceal intussusception that are not diagnosed before or during surgery.

**Fig. 4:**
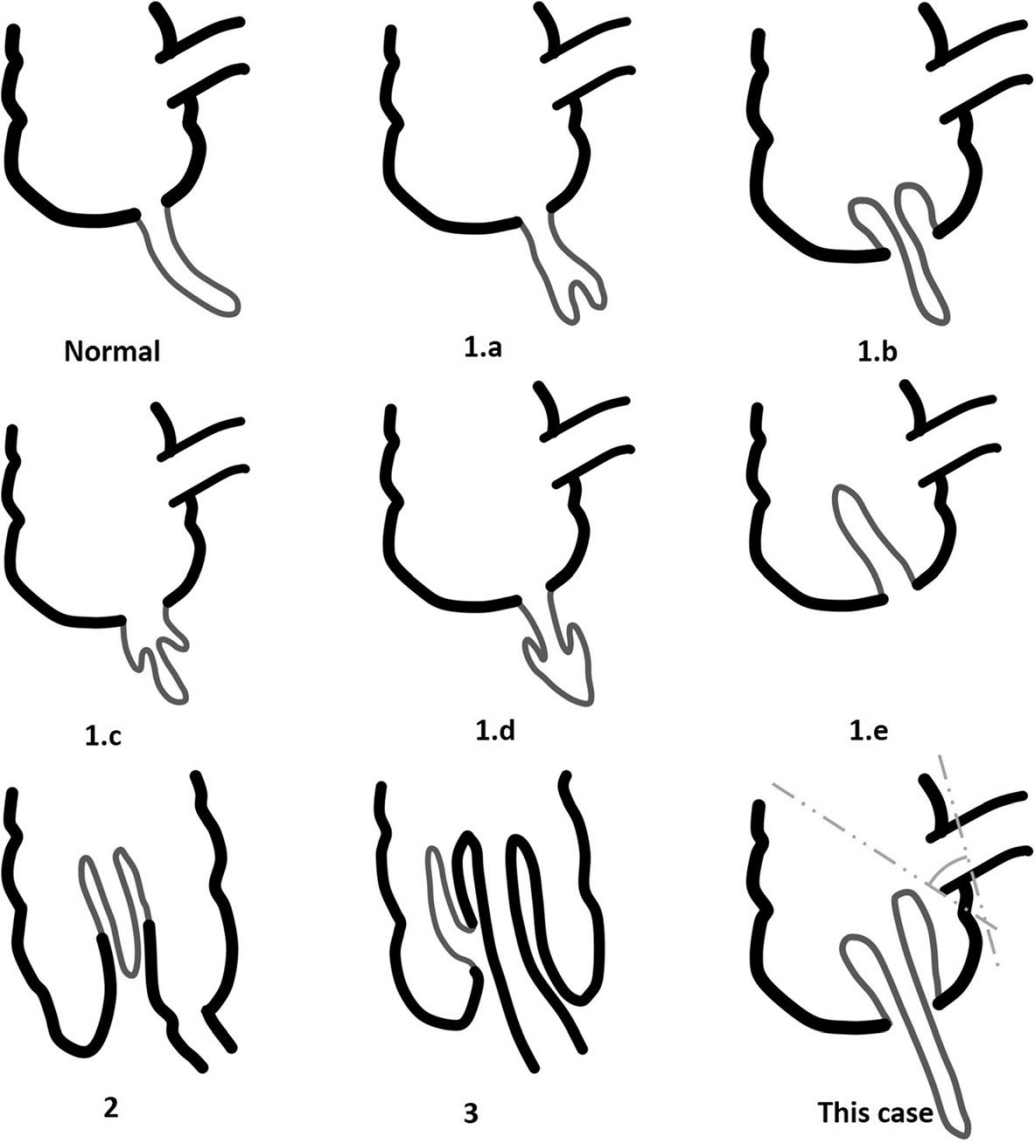
Classification of an appendiceal intussusceptions

If we suspect an appendiceal intussusception before or during surgery, what is the optimal surgery? We can select a surgical procedure based on the classification of appendiceal intussusceptions. Moschcowitz et al. first classified appendiceal intussusceptions, and McSwain expanded the existing classification [1]. Forshall et al. later proposed a comprehensive classification system (Fig. 4) [10]: 1.a – invagination of the appendiceal tip (the intussusceptum) into the proximal appendix (the intussuscipiens); 1.b – invagination beginning at the junction of the appendix and the cecum, in which the appendix is the intussusceptum and the cecum is the intussuscipiens (the most common type; our case); 1.c – invagination beginning along the length of the appendix; 1.d – retrograde intussusception of the proximal appendix into the distal appendix; 1.e – complete invagination of the appendix into the cecum; 2 – compound intussusception (compound or secondary intussusception of the cecocolic type induced by invaginated appendix as a apex with an appendiceal intussusception; 3 – any type of appendiceal intussusception complicated by an ileocolic intussusception; 4 – invagination of an appendiceal mucocele into the cecum. In types 1.a, 1.c, and 1.d, appendectomy is sufficient because the lesion does not include the appendiceal base. On the other hand, in types 1.b and 1.e, the ligated appendiceal base may induce a continuous intussusception following appendectomy alone after reduction of the intussusception. Lipskar el al. reported a case similar to this [4]. Therefore, in types 1.b and 1.e, partial cecectomy should be considered first. In type 2, even though the intussusception can be reduced, it is advisable to perform a partial cecectomy first rather than an appendectomy because of the aforementioned reasons. Following partial cecectomy, another treatment can be considered based on the histopathological results. In type 3, we should identify the origin of the ileocecal intussusception and remove the trigger point. In type 4, if the malignant potential seems to be low or equivocal, the lesion can be managed similarly to types 1.b, 1.e, 2 and 3. However, if malignancy is strongly suspected based on the preoperative evaluation or during surgery, a right hemicolectomy may be considered [8, 9].

In conclusion, surgeons and gastroenterologists can perform safe and reliable treatment by considering appendiceal intussusceptions in cases of appendiceal and cecal diseases. An appropriate surgical treatment can be selected on the basis of the classification of appendiceal intussusceptions. If the cecum should be resected close to the ileocecal valve, it is helpful that we check the angle between the resection line and the ileocecal valve, or exam the IC valve on colonoscopy.

## Abbreviations

CT: Computed tomography
IC: Ileocolic
